# Substance use, risky sexual behaviors, and their associations in a Chinese sample of senior high school students

**DOI:** 10.1186/1471-2458-13-295

**Published:** 2013-04-04

**Authors:** Shenghui Li, Hong Huang, Gang Xu, Yong Cai, Fengrong Huang, Xiuxia Ye

**Affiliations:** 1 School of Public Health affiliated with Shangai Jiaotong University School of Medicine, 227 South Chongqing Road, Shangai, People’s Republic of China; 2 MOE - Shangai Key Laboratory of Children's Environmental Health, Xinhua Hospital, Shangai Jiao Tong University School of Medicine, Shangai, People’s Republic of China; 3 Shangai Municipal Health Bureau, 227 South Chongqing Road, Shanghai, People’s Republic of China

**Keywords:** HIV/STI, Sexual behavior, Substance use, Adolescent, China

## Abstract

**Background:**

Given the higher prevalence of risky sexual behaviors and substance use, adolescents and youths are at risk for HIV. Despite its importance, however, to the best of our knowledge, there are only a few researches on risky behaviors in Chinese adolescents/youths. The present study aimed to describe the prevalence of sexual and substance use behaviors among a Chinese sample of senior high school students. And more specifically, the associations of socio-demographic factors and substance use with risky sexual behaviors were examined in the sample.

**Methods:**

A cross-sectional study was conducted in 10 senior high schools. A total of 2668 senior high school students aged 15.17 to 23.42 years participated in the survey. A self-administrated questionnaire was used to collect information on sexual and substance use behaviors.

**Results:**

The percentages of students who ever had sexual intercourse in lifetime or during last three months were 7.0% and 5.1%, respectively. Among the participants with sexual intercourse during last three months, 42.1% ever had unprotected sexual intercourse and 49.4% had intercourse with two or more partners. Multivariate logistic regression analyses showed that cigarette smoke and illicit drug use were related to unprotected sexual intercourse (defined as “sexual intercourse without condom use”) and younger age of first sexual intercourse was related to multiple-partner sexual intercourse.

**Conclusions:**

HIV/sexual transmitted infection (STI) health education and prevention are necessary among the Chinese adolescents, particularly among those adolescents with experience of sexual intercourse and/or substance use, which has a long-term beneficial to the control of HIV/STI in China.

## Background

Adolescence/youth is often characterized as a stage of increased imitation and exploration with a range of risky behaviors, including risky sexual behaviors and substance use [1-5]. Previous studies revealed that risky behaviors had the characteristic of clustering in one person [1-3,5,6]. For example, risky sexual behaviors (such as multiple sexual partners and unprotect sexual intercourse) among adolescents and youths have been shown to be easy to co-occur with a range of other risky behaviors including alcohol drinking, cigarette smoking, drug use, and violence [1,2,6-8]. In turn, substance use and violence have been implicated in increasing the likelihood for participation in risky sexual behaviors [1,5-7].

A recent report on risky behaviors among American adolescents and youths aged 10–24 years indicated that 4.4% ever had used illicit drugs, 75.0% ever had drunk alcohol, 47.8% ever had sexual intercourse, and 38.5% of currently sexually active adolescents/youths had not used a condom [4]. Researches in other countries similarly found a higher prevalence of risky sexual behaviors and substance use in adolescents and youths [5,7-9]. Given the higher prevalence of risky sexual behaviors and substance use, adolescents and youths aged 15 to 24 years represent approximately 25% of sexually active persons and account for nearly half of new HIV infection each year in the USA [10]. Therefore, adolescents and youths are important subpopulation for sexual-related prevention and intervention efforts.

China is a developing country with a total population of approximately 1.3 billion. Of this 1.3 billion, 24.25% were adolescents and youths aged between 14 and 29 years in 2009 (State statistical bureau of the People’s Republic of China, 2009). With the rapid economic development, sociocultural changes, and globalization, Chinese adolescents and youths are becoming more and more sexually active and easily access to substance use [11-14]. Shangai, the biggest city and metropolises in China, attracts many people of other districts and even other countries to settle and is labeled as a migration city. The ongoing social, cultural, value, and belief mixing may promote the upward trend of exposure to risk behaviors and substance use in adolescents and youths.

Despite its importance, however, to the best of our knowledge, there are only a few researches on risky behaviors in Chinese adolescents/youths. For example, several reports demonstrated that the prevalence of lifetime sexual intercourse was ranged from 1.3% to 4.8% in Chinese senior high school students [11-14]. An earlier survey on drug/psychoactive substance use among adolescents/youths in a south-west province showed the prevalence of substance use were, in rank order, tobacco 4.2%, alcohol 1.6%, NSAID 0.8%, sedative/hypnotic 0.1%, solvents 0.1%, and cannabis 0.1% [15]. A more recent study in Yunnan province found that, among illicit drug user, 64.96% was adolescents and youth (≤18 years, 4.54%, 18–25 years, 60.4%) and 80% of drug users have sexual intercourse experience [16].

During recent years, the growth of the HIV/AIDS epidemic in China is slowing down [17-19]. However, the challenges for HIV/AIDS control are ever bigger than before due to the major shifts in the route of HIV transmission in China: from parenteral to sexual, from high-risk groups to the general population [17-19]. Sexual contact continues to be the major and increasing route of HIV transmission [17-19]. In the context of the efforts to control the transmission of HIV, a full understanding of risky sexual behaviors among general adolescents and youth are of particularly necessary. This necessity is further underscored by the fact that most published studies on risky sexual behaviors mainly focus on high-risk groups. This study filled this knowledge gap by describing the prevalence of sexual risk behavior and substance use engagement by socio-demographic factors among a sample of Chinese high school students. In addition, given unprotected and multiple-partner sexual contact being the most common mode of sexual transmission of HIV infection among adolescents and youth [17-20], we particularly explored the risk factors of ongoing unprotected sexual intercourse and multiple-partner sexual intercourse in the sample.

## Methods

### Sample and procedure

A cross-sectional survey, adopting a two-stage random cluster-sampling design, was conducted in Shangai, the biggest city in China, which is located in eastern coastal area with a total population of approximately 20.8 million in 2008 (State statistical bureau of the People’s Republic of China, 2008) and the highest level of economic development in China. There are twenty districts in Shanghai, 11 being located in urban area, 9 in suburban, and 1 in rural area. From 11 urban districts, two were randomly selected and for every district, 5 senior high schools were randomly selected. All the students in selected schools yielded the sampling frame. Only those students who and their parent(s) both agreed and consented to participate in the study were recruited as eligible sample. Of 2995 students eligible for the study, 2688 (89.1%) returned completed questionnaires. The mean age of the final sample was 17.17 years (SD = 0.72; range, 15.17-23.42 years), 49.9% were males and 50.1% were females.

This study was conducted from April to June of 2008. The research aims were explained to school principals and teachers of the target schools. Permission was obtained from school board to carry out the study, which is the usual practice in China. Informed consent was distributed to all students at the schools, together with written information on the study and another copy of informed consent to be taken home by the children for their parents. After all informed consents had been returned, the survey was implemented during a regular health education class. In the class, an anonymous questionnaire was distributed to all the eligible students. Researchers explained the study purpose to the students and emphasized that participation was voluntary. Every student completed the questionnaire on his/her own desk and nobody else could see his/her responses. Those students who chose not to participate in the study were allowed to engage in other self-directed work, such as reading and writing. All students who did not attend the class were excluded from our survey.

The study protocol was approved by the Ethics Committee of Shanghai Jiaotong University School of Medicine.

### Measure

#### HIV/STI risk behaviors

The Adolescents’ HIV/STI Risk Behaviors Questionnaire (AHRBQ), a self-administrated questionnaire, was used to collect information on adolescents/youths’ behaviors related to sex and substance use. The AHRBQ was derived from our previously established instrument “The Adolescents' Reproductive Health Questionnaire (ARBQ)”. The ARBQ was used to collect information on adolescents' knowledge, attitude, and behaviors concerning HIV/STI, which has been described previously [21]. Based on the 10 items regarding HIV/STI related behaviors in ARBQ, the AHRBQ was developed by adding 9 items according to an updated literature review, qualitative interview in pilot study, and reliability assessment.

The final version of the AHRBQ included 19 items, in addition to demographic items, and the 19 items were conceptually grouped into 3 dimensions: - exposure to sexual behaviors in lifetime (6 items); exposure to sexual behaviors in last three months (9 items); exposure to drug-use behaviors in last three months (4 items) (see Additional file 1). Specific frame for each item of the subscale was noted in Table 1.

**Table 1:** **The response scales for sexual**/**drug**-**use behaviors in the AHRBQ**

	**Response scale**			
	**1**	**2**	**3**	**4**
**Part one** Sexual behaviors in lifetime;				
1. Sexual intercourse in lifetime	yes	no		
2. Gender of the sexual partner lifetime	heterosexual	homosexual	both	
3. Sexual intercourse partners in lifetime	one	two	three	
4. Unprotected sexual intercourse	yes	no		
5. Age of the first sexual intercourse	≤14 years	15-17 years	≥18 years	
6. Age of the first sexual partner	≤14 years	15-17 years	≥18 years	
**Part two** Sexual behaviors in last three months;				
1. Antecedent sexual behaviors with heterosexual partner	no	occasional (1 < time/week)	often (1–2 times/week)	usually (≥times/week)
2. Antecedent sexual behaviors with homosexual partner	no	Occasional	often	usually
3. Sexual intercourse	no	Occasional	often	usually
4. Among these sexual intercourses				
(1) Heterosexual intercourse	Ever had	Never had		
(2) Homosexual intercourse	Ever had	Never had		
(3) Sexual intercourse partners (%)	Ever had	Never had		
(4) Unprotected sexual intercourse	Ever had	Never had		
(5). Sexual intercourse while drunk	Ever had	Never had		
(6) Sexual intercourse with high-risk partners	Ever had	Never had		
**Part three** Substance use behaviors in last three months;				
1. Injection drug use	yes	no		
2. Oral/rhinal drug use	yes	no		
3. Cigarette smoking	no	Occasional	often	usually
4. Alcohol drinking	no	Occasional	often	usually

Antecedent sexual behaviors refers to kiss and petting. Unprotected sexual intercourse refers to the sexual intercourse without condemn use.

High-risk partners refer to people with HIV/AIDS/STI, people with drug use, and people with multiple sexual partners.

The internal consistency of the three dimensions of the AHRBQ was good (Cronbach’s alpha coefficient was ranged from 0.71 to 0.77). The test-retest reliability was evaluated over two week period in a subgroup of our sampled students (n=256) and found to be satisfactory (Intraclass correlation coefficients was 0.85 for the overall questionnaire and ranged from 0.69-0.75 for the dimensions).

#### Socio-economic and demographic characteristics

This section consisted of participant’s gender, age, grade, parents’ education levels and occupation, household income [RMB(yuan)/person/month], parents’ relationship, and family structure (i.e., single parent family, nuclear family, or extended family).

In our study, a family including children, parents, grandfather and/or grandmother, who live together, was defined as extended family; a family including children and parents was defined as nuclear family; and a family only including children and father or mother was defined as single-parent family.

In China, there are no established systems to assess the socioeconomic status (SES) of individual family. Therefore, parental education levels and household income were used as indicators of the family SES.

### Statistical analysis

Statistical descriptions were made by use of the mean, standard deviation and percentages. Demographic differences between males and females were analyzed by Independent-samples *t* test and the Chi-square test. The Chi-square tests were also used to compare differences in prevalence of sexual/drug-use behaviors between males and females.

Multivariate logistic regression analyses were performed to examine the correlating factors of ongoing (defined as having the behavior within last three months) unprotected sexual intercourse (defined as “sexual intercourse without condom use”) and multiple-partner sexual intercourse. All socio-economic and demographic characteristics variables, all substance use variables, and two variables related to sexual intercourse in lifetime (age of the first sexual intercourse and age of the first sexual partner) were entered into the models with the dependent variable designated as “1” if ever had unprotected sexual intercourse/multiple-partner sexual intercourse within last three months and “0” if there was no. Because of the extremely low prevalence of injection drug use and oral/rhinal drug use, a new variable of “Illict drug ues” was created when multivariate logistic regression analyses were performed. The responding scale of “Illict drug ues” is “ever had” if the response to injection drug use or/and oral/rhinal drug use was ever had, and “never had” if the response to injection drug use or/and oral/rhinal drug use were both never had.

The final multivariate models included variables retaining significance after a forward likelihood-ratio stepwise elimination procedure. Statistical tests of regression estimates or OR were based on Wald statistics.

All analyses were performed using the Statistical Program for Social Sciences (SPSS) for Windows, version 12.5. In the presentation of the results, the statistical significance was set at p < 0.05 (two tailed).

## Results

### Socio-economic and demographic characteristics of study sample by gender

Table 2 summarized the socio-economic and demographic outlines of the study sample by gender. 2688 senior high school students aged 17.17±0.72 years participated the survey, 49.9% (n=1324) were males and 50.1% (n=1329) were females.

**Table 2:** **Socio**-**economic and demographic characteristics of study participants**

	**Total (N=2688)**	**Males (N=1324)**	**Females (N=1329)**	***t*/*χ***^***2***^
Age (years, mean±SD)	17.17±0.72	17.20±0.74	17.14±0.70	1.81 ^a^
Family income (%)				0.94 ^b^
Low (<1500)	15.9	15.2	16.4	
Medium (1500–2499)	33.3	33.2	33.6	
High (≥2500)	50.8	51.6	50.0	
Family structure				7.73^b^
Single parent family	7.8	8.1	7.2	
Nuclear family	66.0	63.7	68.5	
Large family	26.2	28.1	24.2	
Father’s education level (%)				10.83** ^b^
Middle school and below	7.6	8.5	6.9	
High school	45.6	42.4	48.7	
College and above	46.8	49.2	44.5	
Mother’s education level (%)				7.08* ^b^
Middle school and below	9.4	9.3	9.6	
High school	50.8	48.4	53.1	
College and above	39.9	42.3	37.3	

Family income was expressed in RMB(yuan)/person/month.

^a^ Independent-samples *t* test.

^b^ K*2 Chi-square Test.

* *P*<0.05.

** *P*<0.01.

No significant differences were observed in age, family income, and family structure between the males and females. However, father’s educational level and mother’s educational level were statistically different between the two groups with the tendency of lower father’s educational levels (*χ2*=10.83, p<0.01), and lower mother’s educational levels (*χ2* =7.08, p<0.05) in females.

#### Sexual behaviors in lifetime

Overall, the percentage of senior high school students who ever had sexual intercourse was 7.0% (n = 188). Compared with females, males had higher prevalence of sexual intercourse (male: 11.0% vs. female: 2.5%, p<0.01). Among the sexually experienced adolescents, 80.5%, 6.0%, and 13.5% reported heterosexual intercourse, homosexual intercourse, and bisexual intercourse, respectively; 39.6% reported two or more sexual partners; 42.4% reported ever had unprotected sexual intercourse; 65.6% reported had the first sexual intercourse under 18 years (≤ 14 years:16.4%; 15–17 years: 49.2%) (Table 3, part one).

**Table 3:** **Prevalence of sexual**/**drug**-**use behaviors of study participants**

	**Total (N=2688)**	**Males (N=1324)**	**Females (N=1329)**	***χ***^***2***^
**Part one** Sexual behaviors in lifetime; n (%)				
1. Sexual intercourse in lifetime				76.95** ^a^
Ever had	188 (7.0)	146 (11.0)	33 (2.5)	
Never had	2500 (93.0)	1178 (89.0)	1296 (97.5)	
2. Gender of the sexual partner lifetime				3.25 ^b^
Heterosexual partner	152 (80.5)	121 (83.0)	31 (70.0)	
Homosexual partner	11 (6.0)	7 (4.7)	4 (15.0)	
Both	25 (13.5)	18 (12.3)	7 (15.0)	
3. Sexual intercourse partners in lifetime				0.62 ^b^
One	113 (60.3)	86 (59.0)	27 (68.4)	
Two	10 (5.3)	8 (5.7)	2 (5.3)	
≥ three	65 (34.4)	52 (35.2)	13 (26.3)	
4. Unprotected sexual intercourse				0.01^a^
Ever had	80 (42.4)	62 (42.5)	18 (52.1)	
Never had	108 (57.6)	84 (57.5)	24 (57.9)	
5. Age of the first sexual intercourse				
≤14 years	31 (16.4)	26 (17.4)	5 (22.0)	3.22 ^b^
15-17 years	92 (49.2)	67 (46.2)	25 (57.1)	
≥18 years	65 (34.4)	53 (36.4)	12 (20.8)	
6. Age of the first sexual partner				0.95 ^b^
≤14 years	30 (15.8)	23 (16.0)	7 (15.0)	
15-17 years	82 (43.6)	66 (45.3)	16 (35.0)	
≥18 years	76 (40.6)	57 (38.7)	19 (50.0)	
**Part two** Sexual behaviors in last three months; n (%)				
1. Antecedent sexual behaviors with heterosexual partner				32.38** ^a^
No/occasional	2548 (94.8)	1225 (92.5)	1294 (97.4)	
Often/usually	140 (5.2)	99 (7.5)	35 (2.6)	
2. Antecedent sexual behaviors with homosexual partner				12.90** ^a^
No/occasional	2642 (98.3)	1309 (98.9)	1306 (98.3)	
Often/usually	46 (1.2)	15 (1.1)	23 (1.7)	
3. Sexual intercourse				
Ever had	137 (5.1)	102 (7.7)	25 (1.9)	46.75** ^a^
Never had	2551 (94.9)	1222 (92.3)	1304 (98.1)	
4. Among these sexual intercourses				
(1) Heterosexual intercourse				17.85** ^a^
Ever had	121 (88.1)	98 (96.0)	17 (68.0)	
Never had	16 (11.9)	4 (4.0)	8 (32.0)	
(2) Homosexual intercourse				2.93 ^a^
Ever had	59 (42.9)	38 (37.1)	14 (56.0)	
Never had	78 (57.1)	64 (62.9)	11 (44.0)	
(3) Sexual intercourse partners (%)				0.76 ^b^
One	69 (50.7)	50 (49.1)	15 (61.5)	
Two	9 (6.7)	7 (7.0)	2 (7.7)	
≥ three	59 (42.7)	45 (43.9)	8 (30.8)	
(4) Unprotected sexual intercourse				0.88 ^a^
Ever had	58 (42.1)	41 (39.7)	13 (52.0)	
Never had	79 (57.9)	61 (60.3)	12 (48.0)	
(5) Sexual intercourse while drunk				4.77* ^a^
Ever had	54 (39.6)	45 (43.9)	5 (20.0)	
Never had	83 (60.4)	57 (56.1)	20 (80.0)	
(6) Sexual intercourse with high-risk partners				1.35 ^a^
Ever had	35 (25.9)	28 (27.3)	4 (16.0)	
Never had	102 (74.1)	74 (72.9)	21 (84.0)	
**Part three** Substance use behaviors in last three months; n (%)				
1. Injection drug use				18.43** ^a^
Ever had	38 (1.4)	29 (2.2)	4 (0.3)	
Never had	2650 (98.6)	1295 (97.8)	1325 (99.7)	
2. Oral/rhinal drug use				13.64** ^a^
Ever had	32 (1.2)	25 (1.9)	5 (0.4)	
Never had	2656 (98.8)	1299 (98.1)	1324 (99.6)	
3. Cigarette smoking				24.75** ^a^
No/occasional	2634 (98.0)	1282 (96.8)	1322 (99.5)	
Often/usually	54 (2.0)	42 (3.2)	7 (0.5)	
4. Alcohol drinking				71.84** ^a^
No/occasional	2580 (96.0)	1231 (93.0)	1321 (99.4)	
Often/usually	108 (4.0)	93 (7.0)	8 (0.6)	

Antecedent sexual behaviors refers to kiss and petting.

Unprotected sexual intercourse refers to the sexual intercourse without condemn use.

High-risk partners refer to people with HIV/AIDS/STI, people with drug use, and people with multiple.

sexual partners.

^a^ 2*2 Chi-square Test.

^b^ K*2 Chi-square Test.

^*^ P<0.05.

^**^P<0.01.

#### Sexual behaviors in last three months

During the three months before the survey, 5.1% (male: 7.7% vs. female: 1.9%, p<0.01) of senior high school students ever had sexual intercourse. Among these sexually experienced participants, 88.1% reported ever had heterosexual intercourse, 42.9% reported ever had homosexual intercourse, 49.4% reported two or more sexual partners, 42.1% reported ever had unprotected sexual intercourse, 39.6% reported ever had sexual 126 intercourse after drunk, and 25.9% reported ever had sexual intercourse with high-risk partners. Among these ongoing sexual behaviors, compared with females, males had higher prevalence of sexual intercourse after drunk (male: 43.9% vs. female: 20.0%, p<0.05). In addition, males had higher frequency of heterosexual antecedent behaviors (male: 7.5% vs. female: 2.6%, p<0.01) (Table 3, part two).

#### Substance use behaviors in last three months

During the three months before the survey, 1.4% of sampled senior high school students reported ever had injection illicit drug use, 1.2% reported ever had oral/rhinal illicit drug use, 2.0% often/usually smoked cigarette, and 4.0% often/usually drunk alcohol. Compared with females, males had higher prevalence of all above substance use (Table 3, part three).

### Risk factors of unprotected sexual intercourse/multiple-partner sexual intercourse of last three months in study sample

Multivariate logistic regression analyses were performed to examine the correlating factors of ongoing unprotected sexual intercourse/multiple-partner sexual intercourse in our study participants.

As indicated in Table 4, after controlling for sociodemographic factors, other substance use behaviors, and two variables related to sexual behaviors in lifetime, two factors in the final model were significantly associated with an increased likelihood of ongoing unprotected sexual intercourse: often/usually cigarette smoke (OR=2.04, p<0.05) and ever had illicit drug use (OR=2.44, p<0.05). For ongoing multiple-partner sexual intercourse, younger age of first sexual intercourse (OR=2.45, p<0.05 for age≤ 14 years, OR=1.94, p<0.05 for age between 15 to 17 years) was the significant risk factor.

**Table 4:** Factors associated with ongoing unprotect sexual intercourse and multiple sexual intercourse during past 3 months by logistic regression models

	**Unprotect sexual intercourse in last three months**		**Multipal sexual partner in last three months**	
	**B**	**OR (95% CI)**	**B**	**OR (95% CI)**
Age		NS		NS
Gender				
Male		NS		NS
Female		1.00		1.00
Father’s educational level				
Middle school and below		NS		NS
High school		NS		NS
College and above		1.00		1.00
Mother’s educational level				
Middle school and below		NS		NS
High school		NS		NS
College and above		1.00		1.00
Family income status				
Low (<1500)		NS		NS
Medium (1500–2499)		NS		NS
High (≥2500)		1.00		1.00
Family structure				
Single parent family		NS		NS
Nuclear/large family		1.00		1.00
Cigarette smoking in last three months				
Often/usually	0.18	2.04 (1.02-4.00) *		NS
No/occasional		1.00		1.00
Drunk in last three months				
Often/usually		NS		NS
No/occasional		1.00		1.00
Illict drug use in last three months				
Ever had	0.24	2.44 (1.15-5.26) *		NS
Never had		1.00		1.00
Age of the first sexual intercourse in lifetime				
≤14 years		NS	0.89	2.45 (1.79-7.08) *
15-17 years		NS	0.68	1.97 (1.24-5.73) *
≥18 years		1.00		1.00
Age of the first sexual partner				
≤14 years		NS		NS
15-17 years		NS		NS
≥18 years		1.00		1.00

Family income was expressed in RMB(yuan)/person/month.

OR, odds ratio.

CI, confidence interval.

NS: no significance.

*P<0.05.

## Discussion

To the best of our knowledge, this study was the first to examine HIV/STI risk behaviors according to three dimensions- sexual behaviors in lifetime, sexual behaviors in last three months, and substance use behaviors in the last three months- in a large sample of urban Chinese high school students. Moreover, we particularly explored the association of substance use and socio-demographic factors with ongoing unprotect sexual intercourse and multiple-partner sexual intercourse.

Overall, our study demonstrated that the proportions of senior high school students who ever had sexual intercourse in lifetime or during last three months were 7.0% and 5.1%, respectively. A number of previous studies revealed that the prevalence of lifetime sexual intercourse was ranged from 1.3% to 4.8% in Chinese senior high school students [11-14,22,23]. In studies of other countries, adolescents who ever had sexual intercourse was 48.7% in the United Sates (aged 10–24 years) [4], 38% in Italy (aged 14–19 years) [24], 17-46% in South African (aged 13–17 years) [25], 11% in Burkina Faso (aged 12–19 years) [26], 18-22% in Nigerian (aged 15–19 years) [27], and 5.1-56.6% in Turkey (aged 16–20 years) [28,29]. The disparity in the prevalence of sexual intercourse among adolescents of different countries may be due to different sample characteristics, different traditional cultural background, and different socioeconomic environment.

Among sexually experienced adolescents, a higher prevalence of unprotected sexual intercourse (42.4% in lifetime and 42.1% in last three months) was found in this sample of urban Chinese students compared their peers of developed countries [4,30,31]. Studies in US, Sweden, and UK showed that unprotected sexual intercourse rate ranged from 14.0% to 38.5% in adolescents [4,30,31]. Previous study indicated that knowledge and awareness about HIV/STI were lower in Chinese adolescents, which may partly account for the higher prevalence of unprotected sexual intercourse in our study sample [11,23]. The present study specially examined the associations between substance use and ongoing unprotected sexual intercourse, where it was shown that substance use, and specifically often/usually cigarette smoking and illicit drug use were strong risk factors for unprotected sexual intercourse, which was in agreement with previous studies [1,5]. A more recent study particularly examined the relationship between substance use and HIV/STI-related sexual risky behaviors among a national sample of sexually active adolescents in American rural settings, where it was similarly found that smoking could increase the likelihood of unprotected sex [32]. There is increasing evidence that illicit drug use may be a risk factor for unprotected sexual intercourse in adolescents by cross-sectional studies [1,8]. A quantitative longitudinal design was implemented to assess causal relationship between illicit drug use and risky sexual behaviors in a sample of gay and bisexual men at 4, 8, and 12 months post-baseline, which indicated that illicit drug use could predict subsequent risky sexual behaviors, such as unprotected sex and multiple-partner sex [33].

The results of our study showed that in our sampled adolescents, 1.4% ever had injection drug use, 1.2% ever had oral/rhinal drug use, 2.0% often/usually smoked, and 4.0% often/usually drunk in last three months. A possible interpretation regarding the mechanisms of association between substance use and high-risk sexual behaviors was that substance use can adversely affect adolescents’ decision-making and result to compromised judgment [34]. In addition, problem behavior theory suggested that problem behaviors (including sexual and substance use activities) tended to cluster and co-occur in adolescents, which was associated with adolescents’ personality, behavior, and the perceived environment [35].

Among the adolescents with experience of sexual intercourse in lifetime or during last three months, 39.7% and 49.4% ever had sexual intercourses with two or more sexual partners, respectively, which was obviously higher prevalent and beyond our expectation. To the best of our knowledge, this study was the first to report Chinese adolescents’ risky sexual behaviors, so, it was unavailable to do comparison with other researches in China. Studies in other countries similarly demonstrated that multiple-partner sex was a common practice in sexual active adolescents [28-30,34-36]. For example, a study in Swiss high school students found that the median number of sexual partners was two in students who had sexual intercourse experience [30]. In the United States, the prevalence of multi-sexual partner was 33.0%-56.0% in adolescents with sexual intercourse experience [36,37]. In other countries, this rate ranged from 40% to 75.2% [28,29]. In high-risk adolescent groups, such as delinquent youth and homeless youth, this rate was extraordinarily high and even reached to above 90.0% [34].

The present study found that younger age of first sexual intercourse was related to an increased tendency of ongoing multiple-partner sexual intercourse. In our sampled adolescents with sexual intercourse experience in lifetime, age of first sexual intercourse was distributed as: ≤14 years (16.4%), 15–17 years (49.5%), and ≥18 years (34.4%). Contrast to a few years ago, age of first sexual intercourse in adolescents of Shanghai urban area were much younger [38,39]. Between 2001 and 2005, the percentage of adolescents who initiated first sexual intercourse <18 years was approximately 20-30% [38,39]. Previous studies suggested that early sexual experience had adverse impact on later sexual activity [37,38]. There was evidence that early sexual intercourse was associated with later substance use, inconsistent condemn use, as well as multiple sex partner [40], which supported the finding of our study. Moreover, a more recent study in men who have sex with men (MSM) found that MSM with a history of childhood sex were more likely to report frequent casual partners and therefore more likely to be HIV positive and to engage in unprotect intercourse [41].

Many factors were associated with adolescents’ initiation of sexual intercourse: age, ethnicity, gender, substance use, economic status, and sociocultural tradition [42,43]. As an Asian country, Chinese society has intrinsic sociocultural values and traditional convention. In Chinese traditional culture, abstain is especially emphasized for unmarried people, especially for females, which is considered to be linked to personal and family honor. However, with the rapid economic development, massive migration, and ongoing acculturation in China, particularly in big Chinese cities (such as Shanghai, Beijing, Guangzhou, and etc.), the traditional conservative value faces challenges. Coming along side an increase in personal freedoms, the spread of the internet, and growing curiosity about overseas norms of behavior, it has contributed to a far more permissive and promiscuous society than was the case in the past. Only a few years ago, the percentage of adolescents who ever had sexual intercourse was ranged from 1.3% to 4.8% in high school students of Beijing, Shanghai, and Guangzhou [11-14,22,23]. Compared to their peers a few years ago, adolescents in big Chinese cities nowadays seemed to be becoming more and more sexually active, which may lead to an increase of sexual behaviors and a shift to younger age of first sexual intercourse [11-14,22,23,38,39].

There are several limitations that should be considered in interpreting these results. Firstly, social-desirability bias and inaccuracy may be existed in answering the questionnaires despite guaranteed anonymity. The second limitation existed in study design. The cross-sectional nature of the study precludes inferences on causality. Thirdly, given the cosmopolitan nature of Shanghai in comparison to the rest of the country, and especially compared to rural China, our findings may not reflect the overall practice in China. Finally, our sample limited our analyses of demographic subgroups (senior high school students in urban area of Shanghai) and can not be generalize to other population.

## Conclusions

In conclusion, our findings provided information regarding Chinese adolescents’ sexual and substance use behaviors. Compared to their peers of many other countries, Chinese adolescents were in a lower level of prevalence of sexual intercourse, however, some risky sexual behaviors, such as unprotected sex and multiple-partner sex, were were more prevalent among those who are sexually experienced. Taking into account a small proportion of youth in China equaling a very large number, we should not take this finding lightly. In addition, we found that substance use and earlier first sexual intercourse could increase the odds of ongoing unprotected sex and multiple-partner sex, respectively. These results suggested that those adolescents with sexual and substance use behaviors seemed to be problem adolescents. Based on the results, we advocated heightened concerns be target the adolescents, particularly those adolescents with sexual and substance use behaviors. Further research is needed to develop effective interventions for this population to reduce sexual and substance use behaviors, which may have a long beneficial to the control of HIV/STI in China.

## Competing interests

There are not any financial competing interests or non-financial competing interests to declare.

## Authors’ contributions

All authors contributed the design of this research. SL drafted the manuscript and has been involved in the interpretation of the data. HH performed statistical analyses. GX, YC, FH, and XY played a major role in the field survey. All authors read and approved the final manuscript.

## Pre-publication history

The pre-publication history for this paper can be accessed here:

http://www.biomedcentral.com/1471-2458/13/295/prepub

## Supplementary Material

Additional file 1 The Adolescents’ HIV/STI Risk Behaviors Questionnaire (AHRBQ). Click here for file
