# Supplementary material for: Substance use, risky sexual behaviors, and their associations in a Chinese sample of senior high school students
Source: BMC Public Health. 2013 Apr 4;13:295. doi: 10.1186/1471-2458-13-295 (PMC3623650; doi:10.1186/1471-2458-13-295)
Supplement: Additional file 1 — The Adolescents’ HIV/STI Risk Behaviors Questionnaire (AHRBQ). [file 1471-2458-13-295-S1.doc]

**The Adolescents’ HIV/STI Risk Behaviors Questionnaire (AHRBQ)**

**Part I Sexual behaviors in lifetime (6 items)**

1. Do you ever have sexual intercourse in lifetime? (if your response is ①, please continue your reply one by one, if your response is ② please skip to Part III )

① yes ② no

1. Is the gender of your sexual partner in lifetime?

① heterosexual ② homosexual ③ both

1. How many sexual intercourse partners do your have in lifetime?

① one ② two ③ ≥ three

1. Do you ever have sexual intercourse without condom use (unprotected sexual intercourse) ?

① yes ② no

1. Do you remember your age of the first sexual intercourse?

① ≤14 years ② 15-17 years ③ ≥18 years

1. Do you remember the age of your first sexual partner?

① ≤14 years ② 15-17 years ③ ≥18 years

**Part II Sexual behaviors in last three months (10 items)**

1. Do you often have antecedent sexual behaviors (such as kiss, petting, and etc) with heterosexual partner in last three months?

① no ② occasional (1 < time/week)

③ often (1-2 times/week) ④ usually (≥times/week)

1. Do you often have antecedent sexual behaviors (such as kiss, petting, and etc) with homosexual partner in last three months?

① no ② occasional (1 < time/week)

③ often (1-2 times/week) ④ usually (≥times/week)

1. Do you often have masturbation in last three months?

① no ② occasional (1 < time/week)

③ often (1-2 times/week) ④ usually (≥times/week)

1. Do you ever have sexual intercourse in last three months ? (if your response is ①, please continue your reply one by one, if your response is ② please skip to Part III )

① yes ② no

Among these sexual intercourses in last three months, do you ever have such as below behaviors?

| 1. Heterosexual intercourse |
| --- |
| ① Ever had ② Never had |
| 1. Homosexual intercourse |
| ① Ever had ② Never had |
| 1. Sexual intercourse partners |
| ① one ② two ③ ≥ three |
| 1. Unprotected sexual intercourse |
| ① Ever had ② Never had |
| 1. Sexual intercourse while drunk |
| ① Ever had ② Never had |
| 1. Sexual intercourse with high-risk partners (people with HIV/AIDS/STI, or drug user, or those with multiple sexual partners) |
| ① Ever had ② Never had |

**Part III Drug-use behaviors in last three months (4 items)**

1. Do you ever have injection drug use in last three months?

① yes ② no

1. Do you ever have oral/rhinal drug use in last three months?

① yes ② no

1. Do you often smoke in last three months?

① no ② occasional (1 < time/week)

③ often (1-2 times/week) ④ usually (≥times/week)

1. Do you often drink alcohol in last three months?

① no ② occasional (1 < time/week)

③ often (1-2 times/week) ④ usually (≥times/week)
